# A prediction model of outcome of SARS-CoV-2 pneumonia based on laboratory findings

**DOI:** 10.1038/s41598-020-71114-7

**Published:** 2020-08-20

**Authors:** Gang Wu, Shuchang Zhou, Yujin Wang, Wenzhi Lv, Shili Wang, Ting Wang, Xiaoming Li

**Affiliations:** 1 Department of Radiology, Tongji Hospital of Tongji Medical College of Huazhong University of Science and Technology, Wuhan, China; 2 Julei Technology, Wuhan, China; 3 Computational Biology, Carnegie Mellon University, Pittsburgh, USA; 4 Department of Medical Ultrasound, Tongji Hospital of Tongji Medical College of Huazhong University of Science and Technology, Wuhan, China

**Keywords:** Infectious diseases, Viral infection

## Abstract

The severe acute respiratory syndrome coronavirus 2 (SARS-CoV-2) has resulted in thousands of deaths in the world. Information about prediction model of prognosis of SARS-CoV-2 infection is scarce. We used machine learning for processing laboratory findings of 110 patients with SARS-CoV-2 pneumonia (including 51 non-survivors and 59 discharged patients). The maximum relevance minimum redundancy (mRMR) algorithm and the least absolute shrinkage and selection operator logistic regression model were used for selection of laboratory features. Seven laboratory features selected in the model were: prothrombin activity, urea, white blood cell, interleukin-2 receptor, indirect bilirubin, myoglobin, and fibrinogen degradation products. The signature constructed using the seven features had 98% [93%, 100%] sensitivity and 91% [84%, 99%] specificity in predicting outcome of SARS-CoV-2 pneumonia. Thus it is feasible to establish an accurate prediction model of outcome of SARS-CoV-2 pneumonia based on laboratory findings.

## Introduction

Most human coronavirus infections are mild. However, several betacoronaviruses can cause serious diseases or even death^1,2^. The mortality rates of severe acute respiratory syndrome coronavirus (SARS-CoV) and Middle East respiratory syndrome coronavirus (MERS-CoV) were 10% and 37% respectively. SARS-CoV-2 is the pathogen for 2019 novel coronavirus disease (COVID-19)^3,4^, which has resulted in thousands of deaths in the world since the beginning of 2020.

The diagnosis of SARS-CoV-2 infection must be confirmed by the real-time reverse transcriptase polymerase chain-reaction (RT-PCR) or gene sequencing of specimens of patients^5,6^. Chest radiograph and laboratory findings are both important for accessing the severity of the disease^7–9^. Critical patients should be admitted to Intensive Care Unit (ICU) of infectious disease hospital, while mild patients could be kept and treated at isolation. It is very important to effectively prioritize resources for patients with the highest risk because of the large number of infected people^10^.

ICU patients and non-ICU patients differed significantly in some blood parameters, including: leukocytes, neutrophils, prothrombin time, D-dimer, total bilirubin (TB), lactate dehydrogenase, high sensitivity cardiac troponin I and procalcitonin^5,7,11^. Ruan et al.^12^ retrospectively analyzed laboratory findings of 68 nonsurvivors and 82 discharged patients, and found significant differences in lymphocytes, platelets, albumin, TB, urea nitrogen, creatinine, myoglobin, C-reactive protein and interleukin-6 between the two groups. These laboratory findings seemed useful in predicting outcome of SARS-CoV-2 infection. However, an advanced prediction model involving multiple laboratory parameters is urgently required to be applied in a clinical-decision support system to improve the predictive and prognostic accuracy.

As a branch of artificial intelligence, machine learning (ML) helps establish accurate prediction model^13–15^. However, there are few publications reporting prediction of the outcome of SARS-CoV-2 pneumonia using ML methods based on laboratory findings. Thus we retrospectively collected laboratory findings of discharged patients and non-survivors. These data were dealt with a ML method similar to radiomics^16,17^. We aim to establish a prediction model of outcome of SARS-CoV-2 pneumonia based on laboratory data.

## Methods

All methods were carried out in accordance with relevant guidelines and regulations.

### Study design and participants

This study was approved by the Ethics Commission of Hospital (TJ-2020-075). Written informed consent was waived by the Ethics Commission of hospital.

The author’s center was the designated hospital for severe and critical SARS-CoV-2 pneumonia. Patients underwent repeated RT-PCR tests to confirm SARS-CoV-2. Laboratory tests for SARS-CoV-2 pneumonia included: blood routine test, serum biochemical (including glucose, renal and liver function, creatine kinase, lactate dehydrogenase, and electrolytes), coagulation profile, cytokine test, markers of myocardial injury, infection-related makers, and other enzymes. Repeated tests were done every 3–6 days for monitoring the patient’s condition.

Oxygen support (from nasal cannula to invasive mechanical ventilation) was administered to patients according to the severity of hypoxaemia. All patients were administered with empirical antibiotic treatment, and received antiviral therapy. Most of patients improved after treatment. However, a few critical patients continued to deteriorate and eventually died.

### Data collection

58 fatal cases of SARS-CoV-2 pneumonia (39 male, median age 66 years) were collected by the electronic medical record system. 68 discharged patients with SARS-CoV-2 pneumonia whose age and gender matched the non-survivors were selected (46 male, median age 66 years). The admission date of these patients was from Feb 16, 2020 to Mar 20, 2020. We reviewed all laboratory findings for each patient. Results of repeated tests were carefully compared to find the greatest deviation from normal value. In general, the greatest number in series of values was recorded. However, for platelets, red blood cell, lymphocytes, hemoglobin, calcium, total protein, albumin, estimated glomerular filtration rate (eGFR), and prothrombin activity (PTA), the minimum was recorded. Laboratory findings at the day of mortality were not used. These recorded laboratory findings were considered as lab features of a patient. A initial data set of 126 patients (non-survivor 58, discharge 68) was thus built.

There were 16 patients who did not have the entire group of laboratory features, thus their data were deleted from the dataset. The remaining data of 110 patients (51 non-survivor, 59 discharge) were analyzed by machine learning.

### Statistical analysis and modeling

First, all the variables were compared between non-survivors and discharged patients using the Mann–Whitney U test for non-normally distributed features or the independent *t* test for normally distributed features^16,17^. Features with *P* < 0.05 were considered significant variables and selected^16,17^. Second, Spearman’s correlation coefficient was used to compute the relevance and redundancy of the features^16,17^. Third, we applied the maximum relevance minimum redundancy (mRMR) algorithm to assess the relevance and redundancy of the features^16,17^. The features were ranked according to their mRMR scores^16,17^. Fourth, the top 15 features with high-relevance and low-redundancy were selected for least absolute shrinkage and selection operator (LASSO) logistic regression model. The LASSO logistic regression model was adopted for further features selection^16,17^. Some candidate features coefficients were shrunk to zero and the remaining variables with non-zero coefficients were finally selected^16,17^. The model was used for calculating signature for each patient. Mann–Whitney U test was used for comparing signature between two groups^16,17^. Receiver operator characteristic (ROC), precision recall curve (PRC) analysis and Hosmer–Lemeshow test were used for further evaluation of model.

The statistical analyses were performed using R software (version 3.3.4; https://www.r-project.org)^16,17^. The following R packages were used: the “corrplot” package was used to calculate Spearman’s correlation coefficient; the “mRMRe” package was used to implement the mRMR algorithm; the “glmnet” was used to perform the LASSO logistic regression model, and the “pROC” package was used to construct the ROC curve^16,17^.

## Results

**Figure 1:**
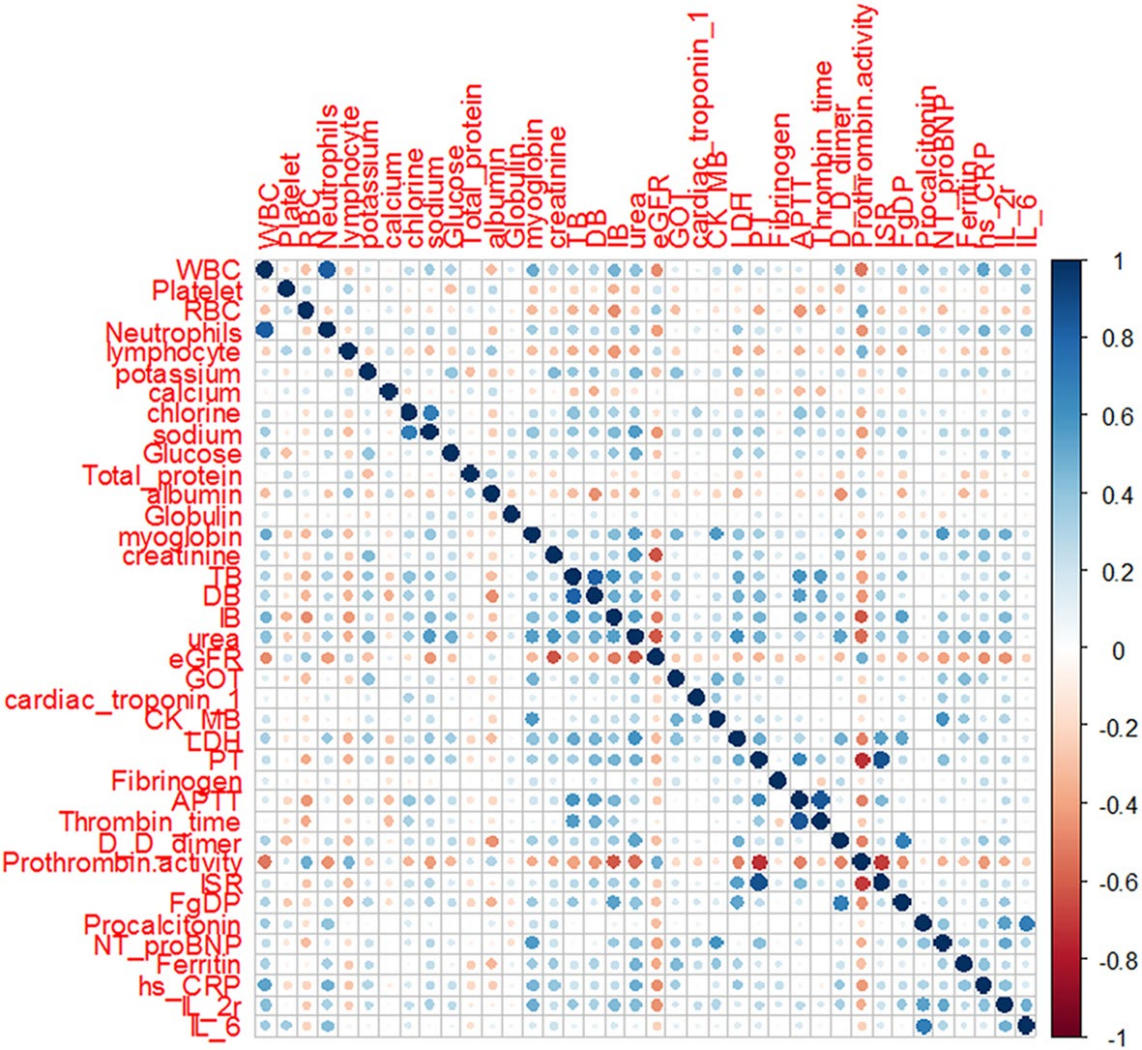
Correlation matrix heatmap of 38 significant features. Spearman’s correlation coefficient was used to compute the relevance and redundancy of the features.

**Figure 2:**
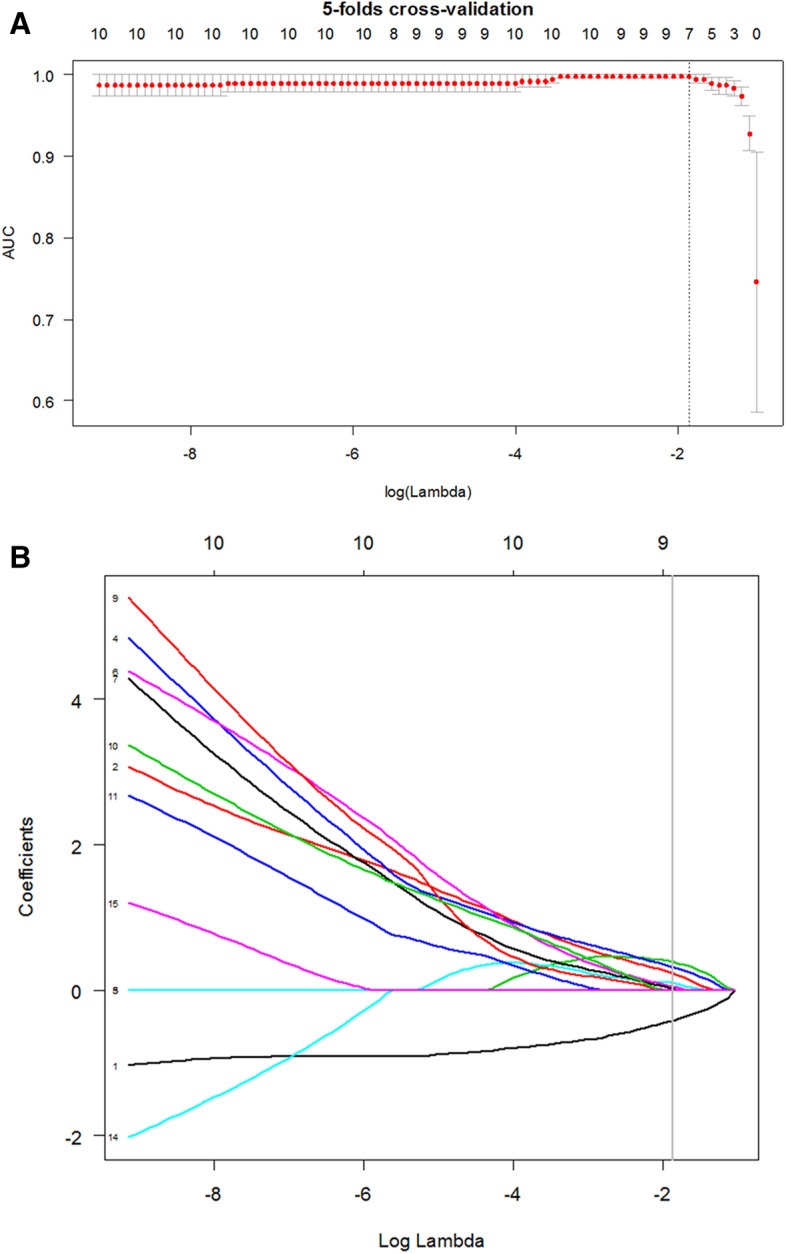
The fivefold cross-validation (**A**) of the least absolute shrinkage and selection operator algorithm for feature selection process. A vertical line was drawn at the optimal value. Some candidate features coefficients were shrunk to zero (**B**) and the remaining seven variables with non-zero coefficients were finally selected.

**Figure 3:**
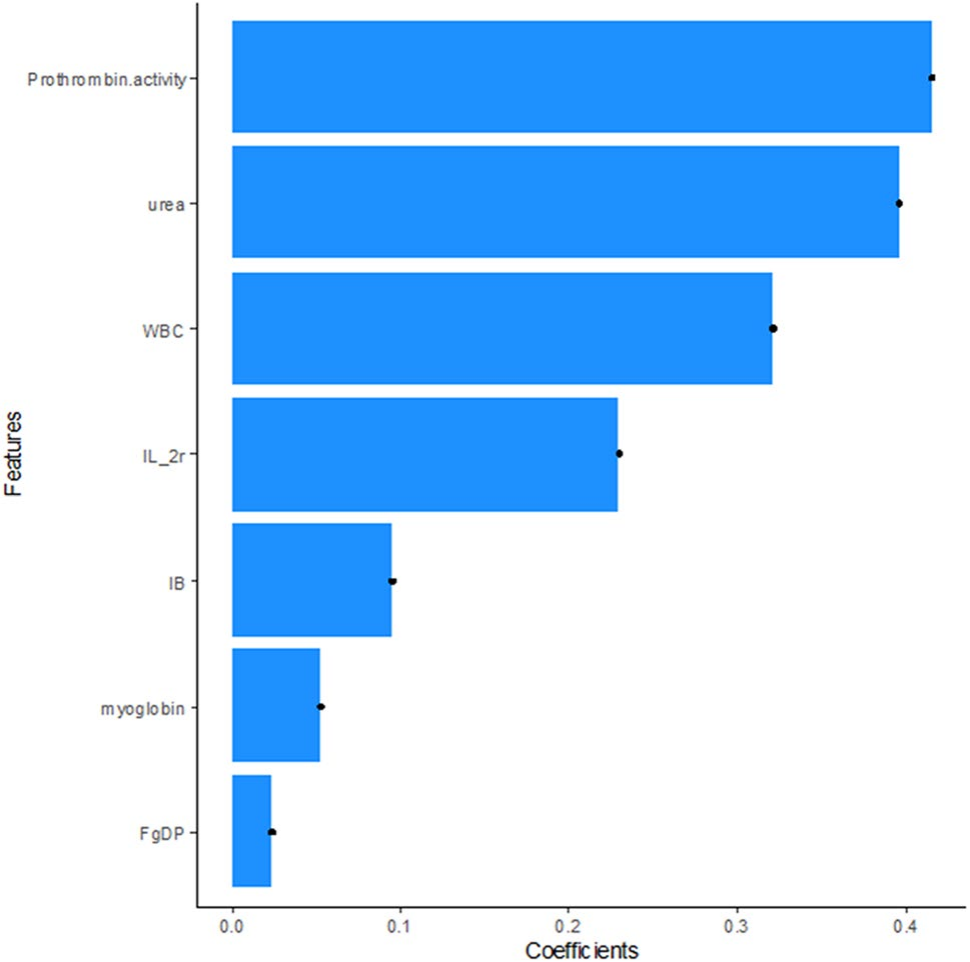
Contribution of the features to the model. The histogram shows the contribution of the seven features with non-zero coefficients. The features are plotted on the y-axis, and their coefficients are plotted on the x-axis.

**Figure 4:**
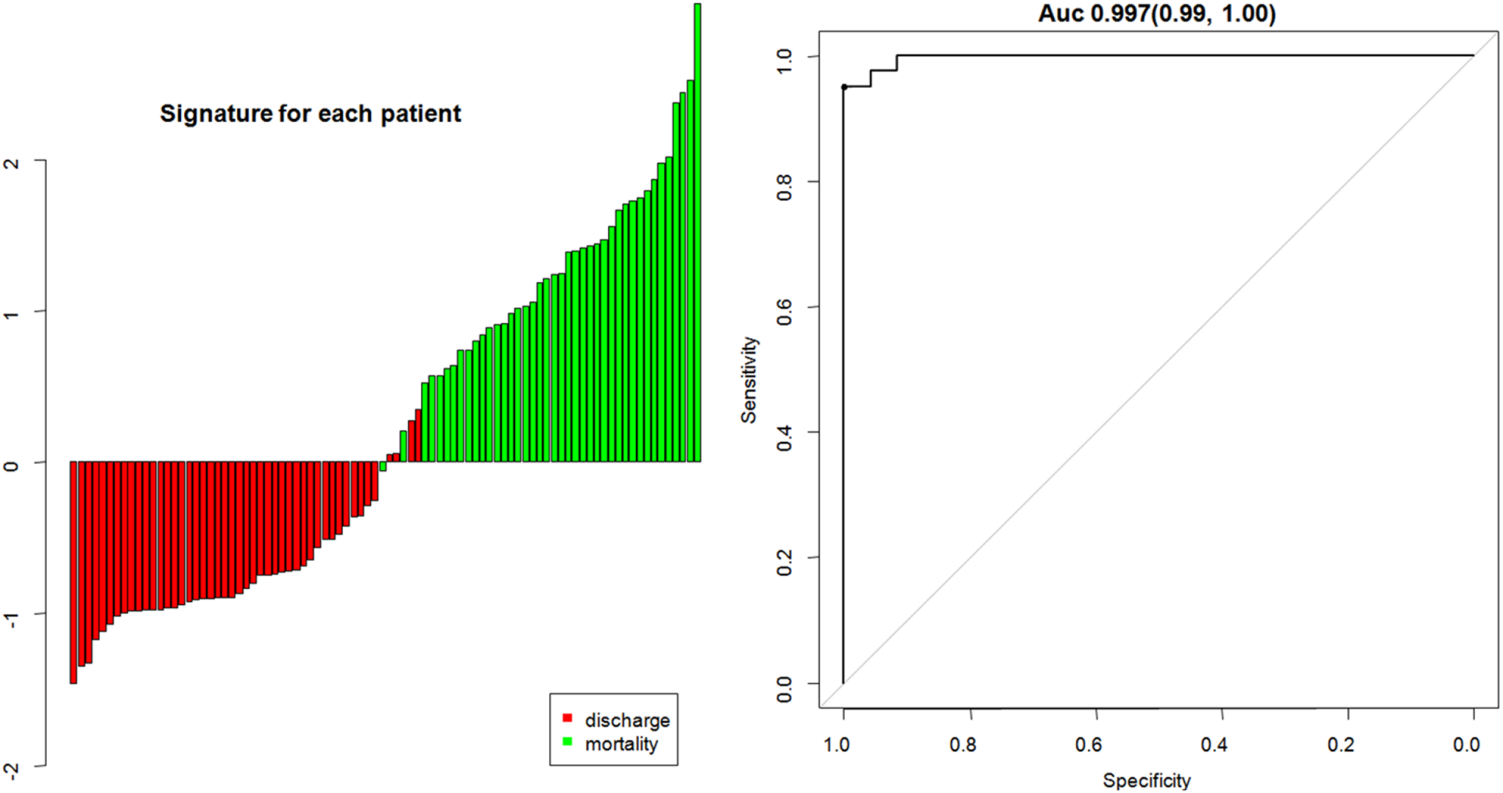
Bar charts of the signature for patients. The red bars indicate the signatures of discharged patients, while the light green bars indicate the signatures of non-survivors. The AUC was 0.997 for the signature.

**Figure 5:**
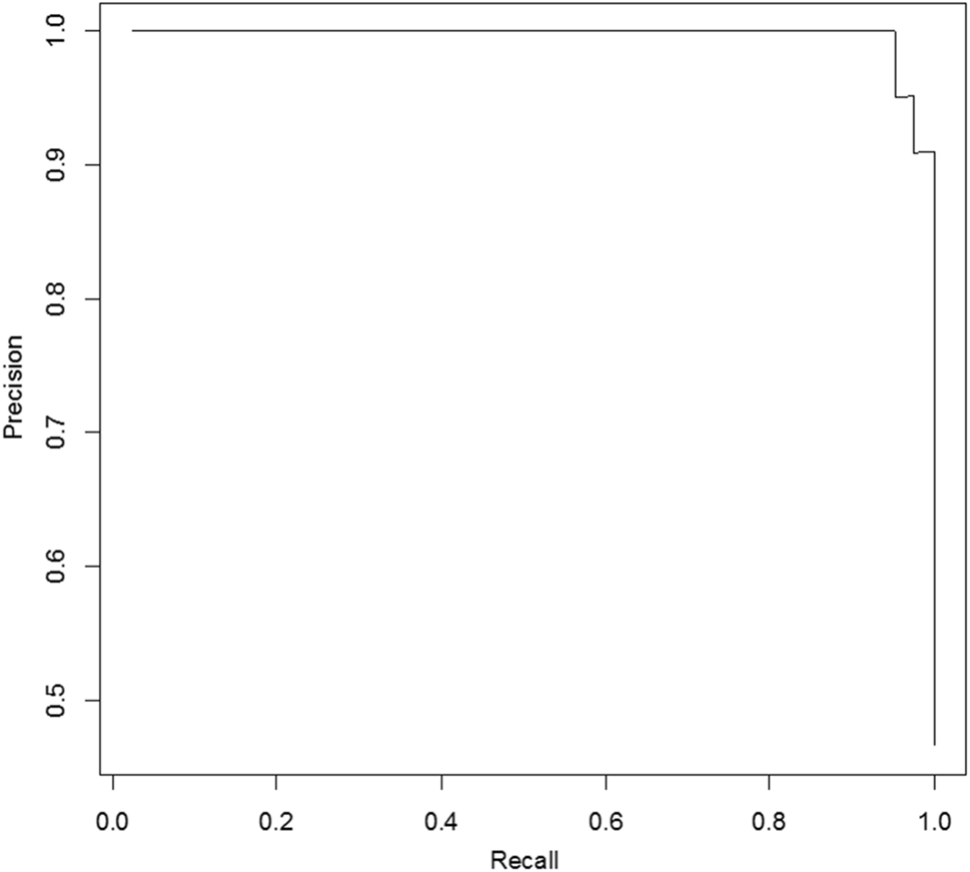
The precision recall curve for the model. The area under precision recall curve was 0.996.

Nine laboratory features were eliminated in the first step of feature selection because of non-significance. The remaining thirty-eight lab features were significantly different between two groups (*P* < 0.05), and then mRMR scores were obtained for them. There were seven features having non-zero coefficients after LASSO algorithm, and were selected for the model. Table 1 shows the fifteen features with the highest mRMR scores. Figure 1 shows the correlation matrix heatmap of the thirty-eight significant features. Figure 2 shows the feature selection process with LASSO algorithm. Figure 3 shows the contribution of the seven features to the model. Figure 4 shows the signatures of all patients, as well as ROC. Figure 5 shows the PRC for the model.

**Table 1:** The fifteen features with higher mRMR scores were selected for the step of LASSO logistic regression.

Rank	Features	mRMR score	Coefficient after LASSO
1	PTA	0.3531	− 0.4148
2	WBC	0.1921	0.3214
3	Urea	0.1867	0.3954
4	IL-2r	0.1773	0.2297
5	IB	0.1249	0.0951
6	Myoglobin	0.1119	0.0526
7	TB	0.1100	0
8	FgDP	0.1073	0.0243
9	hs-CRP	0.1025	0
10	Ferritin	0.0952	0
11	LDH	0.0870	0
12	D-dimer	0.0860	0
13	eGFR	0.0820	0
14	Neutrophils	0.0638	0
15	Sodium	0.0626	0

Some candidate features coefficients were shrunk to zero and the remaining variables with non-zero coefficients were selected.

*mRMR* maximum relevance minimum redundancy, *LASSO* least absolute shrinkage and selection operator, *PTA* prothrombin activity, *WBC* white blood cell, *IL-2r* interleukin-2 receptor, *IB* indirect bilirubin, *TB* total bilirubin, *FgDP* fibrinogen degradation products, *hs-CRP* hypersensitive C-reactive protein, *LDH* lactate dehydrogenase, *eGFR* estimated glomerular filtration rate.

Non-survivors and discharged patients differed significantly in the signature derived from the model (*P* < 0.0001). The AUC was 0.997 [95% CI 0.99, 1.00]. The sensitivity and specificity in predicting outcome of SARS-CoV-2 pneumonia were 98% [93%, 100%] and 91% [84%, 99%] respectively. The area under precision recall curve (AUPRC) was 0.996. Hosmer–Lemeshow test showed good calibration (*P* = 0.95) for the model.

The seven features included in the prediction model were as follows: PTA, urea, white blood cell (WBC), interleukin-2 receptor (IL-2r), indirect bilirubin (IB), myoglobin, and fibrinogen degradation products (FgDP). All features had coefficients of positive number except PTA. PTA and FgDP are from coagulation profile. Urea and IB are from renal and liver function respectively. WBC is from blood routine test. Myoglobin is a marker of myocardial injury. IL-2r is related to immune response. The signatures derived from the model could be positive or negative numbers.

Non-survivors and discharged patients did not differ in age or gender (median age 67 vs. 66, *P* = 0.75; percentage of males, 66% vs. 64%, *P* = 0.66). The comparisons of laboratory findings between non-survivors and discharged patients are shown in Table 2.

**Table 2:** Medians [inter-quartile range] of laboratory findings of patients with SARS-CoV-2 pneumonia were provided in the table.

	Non-survivors	Discharged patients	*P*
Leucocyte (10^9^/L)	11.64 [9.37, 15.61]	5.22 [4.1, 8.79]	< 0.0001
Platelet (10^9^/L)	118 [63, 179]	144.5 [113.5, 227.75]	0.004
Erythrocyte (10^12^/L)	3.48 [2.71, 3.89]	3.76 [3.59, 4.17]	0.001
Neutrophils (10^9^/L)	9.44 [7.36, 12.71]	4.8 [2.45, 7.37]	< 0.0001
Lymphocyte (10^9^/L)	0.5 [0.32, 0.74]	0.70 [0.47, 0.93]	< 0.0001
Hemoglobin (g/L)	115.5 [91, 127]	120 [112.5, 129]	0.16
Potassium (mmol/L)	4.39 [4.11, 5.19]	4.11 [3.70, 4.76]	0.032
Calcium (mmol/L)	2.01 [1.94, 2.10]	2.06 [2.00, 2.13]	0.024
Chlorine (mmol/L)	99.4 [97.2, 105.3]	98 [95.83, 99.4]	0.041
Sodium (mmol/L)	139.55 [135.2, 145.1]	135.55 [133.4, 137.85]	0.017
Glucose (mmol/L)	9.11 [7.62, 13.66]	7.44 [6.46, 9.48]	0.019
Total protein (g/L)	59.9 [56.8, 65.4]	63.9 [59.8, 67.9]	0.030
Globulin (g/L)	37.9 [33.3, 40.7]	32.2 [29.25, 33.85]	< 0.0001
Albumin (g/L)	28 [25.08, 30.95]	32.3 [28.2, 37.2]	0.009
Creatinine (μmol/L)	86 [66, 179.5]	72 [59, 103.5]	0.008
Uric acid (μmol/L)	190.5 [114.5, 309]	188 [148, 362.4]	0.54
Total bilirubin (μmol/L)	17.4 [11.5, 20.4]	11.4 [8.6, 13.4]	< 0.0001
Direct bilirubin (μmol/L)	9.75 [6.4, 14.4]	6.8 [5.35, 9.93]	< 0.0001
Indirect bilirubin (μmol/L)	9.15 [5.45, 11]	7.6 [5.8, 9.35]	0.043
Urea (mmol/L)	14.55 [10, 20.08]	7.85 [6.98, 10.17]	< 0.0001
Estimated glomerular filtration rate (ml/min/1.73 m^2^)	69.3 [41.35, 89.4]	82 [73.4,88.6]	0.008
Glutamic oxaloacetic transaminase (U/L)	43 [24, 104]	37 [21, 57]	0.044
Glutamic-pyruvic transaminase (U/L)	39 [17, 77.25]	38 [24.25, 61.75]	0.70
Myoglobin (ng/mL)	280.6 [152.15, 736.8]	67 [24.45, 129.55]	0.002
High sensitive cardiac troponin I (pg/mL)	202.2 [68.95, 460.18]	30.55 [14.5, 47.6]	< 0.0001
MB isoenzyme of creatine kinase (ng/mL)	5.85 [2.63, 11.93]	1.2 [0.7, 1.9]	0.014
Lactate dehydrogenase (U/L)	490 [358.5, 591]	306.5 [281.5, 368.25]	< 0.0001
Glutamate dehydrogenase (U/L)	16.6 [9, 44]	13.6 [8.23, 25.13]	0.18
Creatine kinase (U/L)	180 [43, 503]	178 [84, 230.5]	0.24
Prothrombin time (s)	16.5 [15.3, 19.4]	14.3 [13.25, 15.83]	< 0.0001
Fibrinogen (g/L)	5.92 [4.82, 6.3]	5.22 [4.59, 5.78]	0.039
Activated partial thromboplastin time (s)	46.4 [42.5, 56]	43.5 [40.65, 46.8]	0.0021
Thrombin time (s)	17.5 [15.7, 20.6]	15.95 [15.13, 17.68]	0.026
D–D dimer (μg/mL)	5.47 [2.73, 12.52]	2.22 [1.82, 2.92]	< 0.0001
Prothrombin activity	62% [55%, 75%]	79% [64%, 91%]	< 0.0001
International standardized ratio	1.3 [1.22, 1.56]	1.08 [0.99, 1.21]	< 0.0001
Fibrinogen degradation products (μg/mL)	29.65 [17.13, 62.65]	6.9 [3.9, 11.5]	< 0.0001
Procalcitonin (ng/mL)	0.97 [0.27, 2.58]	0.16 [0.11, 0.21]	< 0.0001
N-terminal pro-brain natriuretic peptide (pg/mL)	3,375.5 [1491.75, 8,102.75]	963 [522, 1,483.5]	< 0.0001
Ferritin (μg/L)	1,064.5 [814.25, 2,658.5]	826.8 [616.75, 1,481.5]	0.037
Hypersensitive C-reactive protein (mg/L)	142.7 [80.9, 209]	43.1 [22.3, 127.1]	< 0.0001
Interleukin-1β (pg/mL)	5.95 [3.65, 10.28]	5 [3.5, 9.5]	0.17
Interleukin-2 receptor (U/mL)	1,280.5 [1059.25, 1,486.25]	482 [238, 901]	< 0.0001
Interleukin-6 (pg/mL)	157.3 [51.62, 227.3]	75.99 [42.96, 148.80]	< 0.0001
Interleukin-10 (pg/mL)	11.6 [10.25, 15.6]	9.9 [5.3, 14.4]	0.056

Features were compared between non-survivors and discharged patients using the Mann–Whitney U test for non-normally distributed features or the independent *t* test for normally distributed features.

*SARS-CoV-2* severe acute respiratory syndrome coronavirus 2.

### Blood routine test

WBC and neutrophils were significantly higher in non-survivor group versus discharge group. Lymphocyte, platelets and red blood cells were significantly lower in non-survivors. AUC for them were 0.646–0.910.

### Electrolyte

Potassium, chlorine and sodium were significantly higher in non-survivor group versus discharge group. Calcium was significantly lower in non-survivors. AUC for them were 0.634–0.652.

### Serum biochemical test

Glucose and globulin were significantly higher in non-survivor group versus discharge group. Albumin and total protein were significantly lower in non-survivors. AUC for them were 0.649–0.736.

### Renal function

Urea and creatinine were significantly higher in non-survivor group versus discharge group. The eGFR was significantly lower in non-survivors. AUC for them were 0.672–0.907.

### Liver function

Total bilirubin, direct bilirubin, IB and glutamic oxaloacetic transaminase were significantly higher in non-survivor group versus discharge group. AUC for them were 0.647–0.806.

### Coagulation profile

Prothrombin time, activated partial thromboplastin time, D-dimer, international normalized ratio (INR), fibrinogen and FgDP were significantly higher in non-survivor group versus discharge group. PTA was significantly lower in non-survivors. AUC for them were 0.847–0.886.

### Cytokine

IL-2r and IL-6 were significantly higher in non-survivor group versus discharge group. AUC for them were 0.689–0.909.

### Infection-related markers and myocardial injury markers

Procalcitonin, high sensitive C-reactive protein, ferritin and N-terminal pro-brain natriuretic peptide (NT-proBNP) were significantly higher in non-survivor group versus discharge group. Myoglobin, MB isoenzyme of creatine kinase and high sensitive cardiac troponin I were significantly higher in non-survivors. AUC for them were 0.843–0.915.

## Discussion

Non-survivors and discharged patients with SARS-CoV-2 pneumonia differed significantly in thirty-eight laboratory findings. By using machine learning method, we established a prediction model involving seven laboratory features. The model was found highly accurate in distinguishing non-survivors from discharged patients. The seven features selected by artificial intelligence also indicated that dysfunction of multiple organs or systems correlated with the prognosis of SARS-CoV-2 pneumonia.

The SARS-CoV-2 triggers a series of immune responses and induces cytokine storm, resulting in changes in immune components^5,18^. When immune response is dysregulated, it will result in an excessive inflammation, even cause death^7,19^. Excessive neutrophils may contribute to acute lung damage, and are associated with fatality^20^. Higher serum level of IL-2r was found in non-survivors, indicating excessive immune response. In addition, high leukocyte count in SARS-CoV-2 patients may be also due to secondary bacterial infection^21,22^.

Liver injury has been reported to occur during the course of the disease^23,24^, and is associated with the severity of diseases. Increased serum bilirubin level was observed in fatal cases. Acute kidney injury could have been related to direct effects of the virus, hypoxia, or shock^25,26^. Blood urea level continued to increase in some cases. Non-survivors had higher blood urea compared to survivors. Myocardial injury was seen in non-survivors, which was suggested by elevated level of myoglobin. The mechanism of multiple organ dysfunction or failure may be associated with the death of patients with SARS-CoV-2 pneumonia. Some patients with SARS-CoV-2 infection progressed rapidly with sepsis shock, which is well established as one of the most common causes of disseminated intravascular coagulation (DIC)^27^. The non-survivors in our cohort revealed significantly lower PTA compared to survivors. At the late stages of SARS-CoV-2 infection, level of fibrin-related markers (FgDP) markedly elevated in most cases, suggesting a secondary hyperfibrinolysis condition.

A number of laboratory features were compared between non-survivors and discharged patients with SARS-CoV-2 pneumonia. The two groups differed significantly in as many as thirty-eight lab features. However, none of the futures provided adequate accuracy in predicting the outcome of SARS-CoV-2 pneumonia. Thus, a novel prediction model involving multiple features was established in the study. With machine learning methods previously used in radiomics, a prediction model combining seven out of thirty-eight laboratory features was built for predicting the outcome of SARS-CoV-2 pneumonia.

The mRMR algorithm was used for assessing significant features to avoid redundancy between features. The mRMR score of a feature is defined as the mutual information between the status of the patients and this feature minus the average mutual information of previously selected features and this feature^17,28,29^. The top fifteen features with high mRMR scores were selected for the next step of modeling. The least absolute shrinkage and selection operator logistic regression model was used to processing the features selected by mRMR algorithm. LASSO is actually a regression analysis method that improves the model prediction accuracy and interpretability^30^. The signature calculated with the model can be positive or negative number, corresponding with poor and good prognosis respectively. Our results showed that the AUC of the signature was 10–40% higher than that of a single feature.

The modeling process is a black box; however, the choice of variables seems reasonable. PTA can more accurately reflect the coagulation function compared to prothrombin time, and can also reflect the degree of liver injury. Urea is a good index to reflect the degree of renal function damage. WBC can not only reflect immune status, but also secondary infection. IL-2r is an indicator of inflammation and immune response^20^. IB is related to liver function and possible hemolysis. Myoglobin reflects the degree of myocardial injury. The increase of FgDP is related to coagulation disorders including DIC. Thus the current model involves multiple important systems related to prognosis. In consideration of the high accuracy of the model, it can be concluded that liver, kidney, myocardial damage, coagulation disorder and excess immune response all contribute to the outcome of SARS-CoV-2 pneumonia.

It is suitable to start to use this model after three repeated laboratory tests (about 2 weeks after admission), because doctors may have enough data at that time. Lots of laboratory findings are generated in hospitalization. Which are most important for predicting outcome? Our study at least answered such a problem. Seven laboratory features could be used to construct a new signature with the model. The new signature seems more useful than any single feature. We encourage such a simple-to-use model widely used in clinical practice.

Most of clinical factors are not continuous variables (such as underlying disease). We used a machine learning method similar to radiomics, which mainly deals with continuous features. Our study focused on continuous laboratory variables. We had to exclude non-continuous clinical factor with the current machine learning method. By using other methods, a model that involves both continuous variables and category variables can be established. Thus clinical factors raised as significant predictive factors (such as respiratory status or radiological features) could be included in the models. However, there are more than forty laboratory findings in our study, making establishment of model difficult. We felt it necessary to simplify laboratory features. Thus we establish a sub-model based on lab findings. A new lab signature is thus created, and is proved highly valuable. In future study, the signature may be combined with clinical factors to establish a more complex model.

Our study has some limitations. First, this is a single-center retrospective study. Multi-center large-sample studies are required to validate our prediction model. Second, our model may not be directly used in other centers. However, they could easily establish a prediction model using their own data with machine learning method. Third, some patients who did not have all the lab findings were excluded. Selection bias must be present due to patients exclusion. Other studies with more strict design were thus required to reveal the bias. Fourth, statistical approach conducted in this study is not perfect. As LASSO was used for 15 variables, 150 or more patients were needed. More patients should be collected in future study.

In conclusion, it is feasible to establish a accurate prediction model of outcome of SARS-CoV-2 pneumonia based on laboratory findings. Injury of liver, kidney and myocardium, coagulation disorder and excess immune response all correlate with the outcome of SARS-CoV-2 pneumonia.

## Data Availability

After publication, the data will be made available to others on reasonable requests to the corresponding author.

## References

[1] Drosten C (2003). Identification of a novel coronavirus in patients with severe acuterespiratory syndrome. N. Engl. J. Med..

[2] Zaki AM, Boheemen S, Bestebroer TM, Osterhaus AD, Fouchier RA (2012). Isolation of a novel coronavirus from a man with pneumonia in Saudi Arabia. N. Engl. J. Med..

[3] Phelan AL, Katz R, Gostin LO (2020). The novel coronavirus originating in Wuhan, China: challenges for global health governance. JAMA.

[4] Li Q (2020). Early transmission dynamics in Wuhan, China, of novel coronavirus-infected pneumonia. N. Engl. J. Med..

[5] Huang C (2020). Clinical features of patients infected with 2019 novel coronavirus in Wuhan, China. Lancet.

[6] Zhu N (2020). A novel coronavirus from patients with pneumonia in China, 2019. N. Engl. J. Med..

[7] Wang D (2020). Clinical characteristics of 138 hospitalized patients with 2019 novel coronavirus-infected pneumonia in Wuhan, China. JAMA.

[8] Bernheim A (2020). Chest CT findings in coronavirus disease-19 (COVID-19): relationship to duration of infection. Radiology.

[9] Fang Y (2020). Sensitivity of chest CT for COVID-19: comparison to RT-PCR. Radiology.

[10] General Office of the National Health Commission of China. Diagnosis and treatment protocol for 2019-nCoV. 5th ed. Beijing, China: National Health Commission of China (2020).

[11] Yang X (2020). Clinical course and outcomes of critically ill patients with SARS-CoV-2 pneumonia in Wuhan, China: a single-centered, retrospective, observational study. Lancet Respir. Med..

[12] Ruan Q, Yang K, Wang W, Jiang L, Song J (2020). Clinical predictors of mortality due to COVID-19 based on an analysis of data of 150 patients from Wuhan, China. Intensive Care Med..

[13] Shiri I (2020). Next-generation radiogenomics sequencing for prediction of EGFR and KRAS mutation status in NSCLC patients using multimodal imaging and machine learning algorithms. Mol. Imaging Biol..

[14] Matsuzaka Y (2020). Prediction model of aryl hydrocarbon receptor activation by a novel QSAR approach, deepSnap-deep learning. Molecules.

[15] Katić K, Li R, Zeiler W (2020). Machine learning algorithms applied to a prediction of personal overall thermal comfort using skin temperatures and occupants’ heating behavior. Appl. Ergon..

[16] Jiang M (2020). Nomogram based on shear-wave elastography radiomics can improve preoperative cervical lymph node staging for papillary thyroid carcinoma. Thyroid.

[17] Zhang P (2019). T2-weighted image-based radiomics signature for discriminating between seminomas and nonseminoma. Front. Oncol..

[18] Qin C (2020). Dysregulation of immune response in patients with COVID-19 in Wuhan, China. Clin. Infect. Dis..

[19] Mahallawi WH, Khabour OF, Zhang Q, Makhdoum HM, Suliman BA (2018). MERS-CoV infection in humans is associated with a pro-inflammatory Th1 and Th17 cytokine profile. Cytokine.

[20] Channappanavar R, Perlman S (2017). Pathogenic human coronavirus infections: causes and consequences of cytokine storm and immunopathology. Semin. Immunopathol..

[21] Chen N (2020). Epidemiological and clinical characteristics of 99 cases of 2019 novel coronavirus pneumonia in Wuhan, China: a descriptive study. Lancet.

[22] Guan W (2020). Clinical characteristics of 2019 novel coronavirus infection in China. N. Engl. J. Med..

[23] Tang N, Li D, Wang X, Sun Z (2020). Abnormal coagulation parameters are associated with poor prognosis in patients with novel coronavirus pneumonia. J. Thromb. Haemost..

[24] Xu L, Liu J, Lu M, Yang D, Zheng X (2020). Liver injury during highly pathogenic human coronavirus infections. Liver Int..

[25] Estenssoro E (2010). Pandemic 2009 influenza A in Argentina: a study of 337 patients on mechanical ventilation. Am. J. Respir. Crit. Care Med..

[26] Li K (2020). The clinical and chest CT features associated with severe and critical COVID-19 pneumonia. Investig. Radiol..

[27] Abe T (2020). Complement activation in human sepsis is related to sepsis-induced disseminated intravascular coagulation. Shock.

[28] Lin X, Li C, Ren W, Luo X, Qi Y (2019). A new feature selection method based on symmetrical uncertainty and interaction gain. Comput. Biol. Chem..

[29] Wang J (2017). Machine learning-based analysis of MR radiomics can help to improve the diagnostic performance of PI-RADS v2 in clinically relevant prostate cancer. Eur. Radiol..

[30] Sauerbrei W, Royston P, Binder H (2007). Selection of important variables and determination of functional form for continuous predictors in multivariable model building. Stat. Med..

